# Safety of over Twelve Hundred Infant Male Circumcisions Using the Mogen Clamp in Kenya

**DOI:** 10.1371/journal.pone.0047395

**Published:** 2012-10-17

**Authors:** Marisa R. Young, Robert C. Bailey, Elijah Odoyo-June, Tracy E. Irwin, Walter Obiero, Dedan O. Ongong'a, Jacinta A. Badia, Kawango Agot, Sherry K. Nordstrom

**Affiliations:** 1 Department of Epidemiology and Biostatistics, University of Illinois at Chicago School of Public Health, Chicago, Illinois, United States of America; 2 Nyanza Reproductive Health Society, Kisumu, Kenya; 3 Department of Obstetrics and Gynecology, University of Illinois at Chicago College of Medicine, Chicago, Illinois, United States of America; 4 Department of Surgery, Nyanza Provincial General Hospital, Kisumu, Kenya; 5 CARE International, Kisii, Kenya; 6 Impact Research and Development Organisation, Kisumu, Kenya; University of Massachusetts Medical School, United States of America

## Abstract

**Background:**

Several sub-Saharan African countries plan to scale-up infant male circumcision (IMC) for cost-efficient HIV prevention. Little data exist about the safety of IMC in East and southern Africa. We calculated adverse event (AE) rate and risks for AEs associated with introduction of IMC services at five government health facilities in western Kenya.

**Methods:**

AE data were analyzed for IMC procedures performed between September, 2009 and November, 2011. Healthy infants aged ≤2 months and weighing ≥2.5 kg were eligible for IMC. Following parental consent, trained clinicians provided IMC services free of charge under local anesthesia using the Mogen clamp. Odds ratios and 95% confidence intervals were used to explore AE risk factors.

**Findings:**

A total of 1,239 IMC procedures were performed. Median age of infants was 4 days (IQR = 1, 16). The overall AE rate among infants reviewed post-operatively was 2.7% (18/678; 95%CI: 1.4, 3.9). There was one severe AE involving excision of a small piece of the lateral aspect of the glans penis. Other AEs were mild or moderate and were treated conservatively. Babies one month of age or older were more likely to have an AE (OR 3.20; 95%CI: 1.23, 8.36). AE rate did not differ by nurse versus clinical officer or number of previous procedures performed.

**Conclusion:**

IMC services provided in Kenyan Government hospitals in the context of routine IMC programming have AE rates comparable to those in developed countries. The optimal time for IMC is within the first month of life.

## Introduction

Male circumcision (MC) has been practiced for cultural and religious reasons for more than 4,000 years [1]. Although MC has been associated (often spuriously) with various behaviors and health outcomes over the centuries, recent epidemiological and biological evidence demonstrates MC confers several health benefits. These include: reduced risk of heterosexual HIV acquisition in men by 50–60%, reduced risk of genital ulcer disease and ulcerative sexually transmitted infections, and reduction in oncogenic HPV incidence and penile cancer [2]–[6]. Benefits to female sexual partners of circumcised men include reduced risk of bacterial vaginosis, *trichomonas vaginalis*, and cervical cancer [7]–[10]. In addition to these benefits, boys circumcised in infancy have reduced risk of urinary tract infections in the first year of life and avoid pathologies of the foreskin, including phimosis, paraphimosis and balanitis that may be medical indications for MC [1], [11].

Global prevalence of MC varies widely, ranging from greater than 80% in the Middle East, North Africa and West Africa to less than 20% in Europe, most of Asia and Latin America [12]. In East and southern Africa, ethnicity is a major determinant of circumcision practices. Among circumcising communities in this region, MC is typically performed during adolescence [12]. In Kenya, an estimated 86% of men aged 15–49 are circumcised, the vast majority during adolescence [13]. Infant circumcision is little-known and infrequently practiced in Kenya and throughout East and southern Africa [12].

Following the results of three randomized controlled trials demonstrating the protective effect of MC against HIV acquisition, the WHO and UNAIDS recommended scale-up of MC services in areas with low prevalence of MC, high HIV burden and where the disease is primarily transmitted through heterosexual sex [14]. Roll-out of adolescent and adult MC services is now underway in 14 countries in East and southern Africa [15]. To date, the focus of research and program implementation has been primarily on adult MC [16]–[18]. In comparison to adolescent and adult MC, the circumcision of an infant is less expensive, safer, easier to perform and likely to reduce the chance of risk compensation [19]–[21]. Two recent cost-effectiveness analyses have found infant male circumcision (IMC) is cost-saving for HIV prevention under a wide variety of conditions, including those that prevail in many African nations [22], [23]. As MC is scaled-up, governments are likely to shift efforts towards IMC for long-term, sustainable HIV prevention along with other benefits. If IMC programs are to be effectively rolled-out, providing safe surgical services will be of paramount importance. This paper reports rate, type and severity of adverse events (AE) measured as part of provision of IMC services in Nyanza Province, Kenya where IMC is not traditionally practiced.

## Methods

In partnership with the Kenyan Ministry of Health, we introduced infant male circumcision services in five Government health facilities in three Districts in Nyanza Province, western Kenya. The data for this analysis come from two sources: (1) a case/control study [24] examining factors associated with parental acceptance of IMC services where infants were actively followed-up postoperatively (research infants), and (2) routine monitoring of IMC services where post-operative follow-up was passive (non-research infants). In the research study, “case” parents were those who accepted IMC for a son and “control” parents had declined IMC services for an eligible son. We used safety data from babies who were circumcised. Information on demographic characteristics of parents and parental satisfaction with IMC was recorded for research infants but not for non-research infants. The following information was collected as part of routine monitoring of IMC services for all infants: date and location of procedure, name of parent/guardian, weight of baby, date of birth of baby, IMC provider and cadre, date of follow-up (if any), and type and severity of any intra-operative or post-operative adverse event. IMC services were available to term male neonates and infants, aged ≤2 months, weighing ≥2.5 kg, generally healthy (eg: absence of fever and jaundice) and without an anomaly of the genitals. All mothers (and fathers, if present) were counseled on the benefits and risks of IMC and provided written informed consent for the procedure prior to surgery.

IMC services were provided on a voluntary basis to all infants free of charge by a trained nurse or clinical officer (similar to a physician's assistant) using the Mogen clamp method. A dorsal penile nerve block of 0.15 mg per kg of 2% lidocaine, diluted with an equal amount of water for injection, was administered for intra-operative analgesia. Parents were given verbal and written instructions on post-operative wound care in their language of choice (English, Kiswahili or DhoLuo), a 50 ml bottle of petroleum jelly to apply to the wound, a 50 ml bottle of acetaminophen (paracetamol) for post-operative analgesia, and one disposable diaper. All parents were asked to return to the health facility with the infant three to four days following IMC to assess progress of wound healing. Research participants were given a transport stipend (approximately 1.50 USD) upon return to the health facility for the post-operative review and were actively followed-up if they did not return. Active follow-up included calls to the parents, attempted tracing, and home visits to review the wound post-operatively. Non-research infants were not given a stipend and were not called or traced if they failed to return for review. We used a standardized data collection form for documenting AEs that was adapted from our earlier trial of adult male circumcision for HIV prevention in Kisumu [2] and was completed by an IMC-trained clinician. All parents were given the telephone number for an IMC hotline, staffed by an IMC-trained clinician and instructed to call with any questions or concerns.

This analysis is largely descriptive in nature. We report medians, inter-quartile ranges (IQR) and proportions, as appropriate. Differences in AE rates by provider type, provider experience level, and age of baby were computed using odds ratios (ORs). Data were entered into Microsoft Access 2007 (Microsoft Corporation, Seattle, WA, USA) and imported into Stata/IC 12.1 for Mac (StataCorp, College Station, TX, USA) for analysis. The University of Illinois at Chicago Institutional Review Board and the Kenyatta National Hospital Ethics and Research Committee provided ethical approval for the research study. Data on infant safety in non-research participants was collected according to Government of Kenya monitoring and evaluation guidelines for routine IMC services.

## Results

Between September 1, 2009 and November 29, 2011, 1,261 babies were registered for IMC at the five facilities where the procedure was offered. Of these, 22 infants (2%) were excluded from surgery due to contraindications discovered after entry into the surgical register: eleven with penile anomaly (nine hypospadias, one epispadias, one micropenis), five with dense foreskin adhesions, two with fever, two with impetigo, one with severe phimosis, and one missing reason. All but the last of these were referred to a pediatric surgeon who was a co-investigator in the study (DO) or to a nurse at the health facility where the IMC took place.

Fifty one providers performed 1,239 IMC procedures (three quarters of procedures were provided by 10 clinicians). Three hundred and eight infants (25%) were research infants and the remaining 931 infants were non-research infants. The median age of babies circumcised was four days (IQR 1–16). Among research infants, 294 of 308 infants (95%) were reviewed post-operatively. The post-operative review rate among non-research infants was 41% (384/931). Other characteristics of research and non-research infants are listed in Table 1.

**Table 1:** Characteristics of research and non-research IMC procedures.

	Research Infants (active follow-up) n = 308		Non-research Infants (passive follow-up) n = 931	
	n	%	n	%
**Post-operative follow up**				
Yes	294	95.5	384	41.3
No	14	4.5	547	58.8
**Age, days [median (IQR)]**	7 (1, 26)		4 (1, 10)	
**Provider type**				
Nurse	233	75.7	590	63.4
Clinical Officer	75	24.4	326	35.0
Missing	0	0.0	15	1.6
**Experience level of provider**				
<10 procedures	73	23.7	221	23.7
10+ procedures	234	76.0	674	72.4
Missing	1	0.3	36	3.9
**Satisfaction with IMC at post-operative visit**				
Very satisfied	282	95.9	N/A	
Somewhat satisfied	11	3.7	N/A	
Somewhat dissatisfied	1	0.3	N/A	
Very dissatisfied	0	0.0	N/A	
**Would circumcise son again** ‡				
Yes	293	99.7	N/A	
No	0	0.0	N/A	
Missing	1	0.3	N/A	

IQR, Inter-quartile range; N/A, Not applicable.

‡ Question wording: “If you were to do it again, would you circumcise your baby?”

Most post-operative reviews (96%) occurred within one week of surgery. The remaining 4% occurred up to 45 days following IMC. Seven research infants had an IMC-related adverse event (AE rate 2.4%, 95%CI: 0.6, 4.1). One research infant had two AEs, for a total of eight adverse events in this group. Among non-research infants returning for review, the AE rate was 2.9% (11/384, 95%CI: 1.2, 4.5). Therefore, there were a total of 19 AEs detected in 18 unique infants out of 678 infants reviewed post-operatively (Total AE rate 2.7%, 95%CI: 1.4, 3.9). Out of the total 1,239 infants circumcised, the AE rate was 1.5% (95%CI: 0.8, 2.1). We had one severe AE involving excision of a small piece of the dorso-lateral aspect of the glans penis. Other events were classified as mild or moderate and treated conservatively (see Table 2). These included: intra-operative or post-operative bleeding arrested with pressure (n = 7) or sutures (n = 3), post-operative abrasion (n = 3), and wound infection (n = 5).

**Table 2:** Type and frequency of adverse events and other events.

	Research Infants n = 308	Non-research Infants n = 931
IMC-related Adverse Events	n (%)	n (%)
**Severe AE**		
Damage to the glans	0 (0.0)	1 (0.1)
**Mild/Moderate AE**		
Intra-operative bleeding - suture required	1 (0.3)	2 (0.2)
Intra-operative bleeding - resolved with pressure	2 (0.6)	2 (0.2)
Post-operative bleeding - resolved with pressure	1 (0.3)	2 (0.2)
Infection	2 (0.6)	3 (0.3)
Meatal abrasion	2 (0.6)	1 (0.1)
*Sub-total:*	8 (2.6)	11 (1.2)
**Other Events**		
Too little foreskin removed	3 (1.0)	6 (0.6)
Impetigo/rash not involving genitals	1 (0.3)	2 (0.2)
Post-operative fever without other symptoms, not IMC related	0 (0.0)	2 (0.2)
*Sub-total:*	4 (1.3)	10 (1.1)

AE, adverse event; IMC, infant male circumcision.

AEs were more common in older babies; 7/205 babies aged one month or older (3.4%) experienced an adverse event, in comparison to 11/1,007 babies (1.1%) less than one month of age (OR = 3.20, p = 0.02). There was no difference in AE rate by type of provider or experience level of provider (Table 3).

**Table 3:** Adverse event rate and risk factors for adverse events in research and non-research circumcisions.

	Research Infants (active follow-up) n = 308					Non-research Infants (passive follow-up) n = 931					Combined N = 1,239				
AE rate (reviewed post-op)	7/294 (2.4%; 95%CI: 0.6, 4.1)					11/384 (2.9%; 95%CI: 1.2, 4.5)					18/678 (2.7%; 95%CI: 1.4, 3.9)				
AE rate (all procedures)	7/308 (2.3%; 95%CI: 0.6, 3.9)					11/931 (1.2%; 95%CI: 0.5, 1.9)					18/1239 (1.5%; 95%CI: 0.8, 2.1)				
	AEs	IMCs	%	OR	p	AEs	IMCs	%	OR	p	AEs	IMCs	%	OR	p
**Provider type**				0.80	0.79				0.97	0.96				0.97	0.96
Nurse	5	233	2.1			7	590	1.2			12	823	1.5		
Clinical Officer	2	75	2.7			4	326	1.2			6	401	1.5		
**Experience level**				2.46	0.25				1.15	0.84				1.56	0.38
<10 procedures	3	73	4.1			3	221	1.4			6	294	2.0		
10+ procedures	4	234	1.7			8	674	1.2			12	908	1.3		
**Baby's age**				2.72	0.20				3.27	0.06				3.20	0.02
Age 30+ days	3	68	4.4			4	137	2.9			7	205	3.4		
Age <30 days	4	240	1.7			7	767	0.9			11	1,007	1.1		

AE, adverse event; CI, confidence interval; IMC, infant male circumcision; OR, odds ratio.

Nearly all (282/294, 96%) parents of research infants reported being ‘very satisfied’ with IMC services received. All parents of research infants returning for review who were asked said they would choose circumcision for their son, if they could do it again. We did not ask non-research parents about satisfaction or the decision to circumcise again.

## Discussion

There are few published reports on safety of IMC in developing country settings [12]. The available data show adverse event rates associated with IMC vary widely by setting, type of provider, method used and classification of what constitutes an AE. A recent review found that the median AE rate following neonatal and infant MC was 1.5% (range 0%–16%) among 16 prospective studies from 12 countries [19]. The median rate of serious AE was 0% (range 0%–2%). The review did not include any studies in which the Mogen clamp method was used. We are aware of only one other study reporting AE rates from East Africa, where IMC is rarely performed [25]. In that Tanzanian study, 368 infants were circumcised using the Plastibell device with an overall AE rate of 2.8% and no serious AEs. Our observed AE rate of 2.4% among research infants and 2.9% among non-research infants is consistent with the lower range of AEs reported from studies conducted in the developing world [25]–[29]. Lack of consistency in ascertainment and definition of AEs contributes to the wide range of published AE rates. For example, we elected not to include cases of too little foreskin removed (n = 9) in our adverse event calculations because this is not a medical adverse event, *per se*. Others have chosen to include this type of event, because re-breaking of adhesions and corrective surgery may be necessary in the future. If we included these cases in our count of AEs, the overall AE rate among those returning for post-operative review would be 4.0% (27/678; 95%CI: 2.5, 5.5).

The wide variation in AE rates reported in the literature may be due to differences in experience and training of provider, traditional versus medical IMC, device used, location of procedure, and age of infant. In this study, IMC was provided in a health care setting by trained medical providers (nurses or clinical officers) using sterile equipment on neonates and young infants (≤2 months of age): factors that likely contributed to our low observed adverse event rate. We found no difference in AE rates by provider type, a finding that supports the inclusion of trained nurses as IMC providers. Infants one month of age or older had over three times the odds of experiencing an AE and this result was statistically significant (p = 0.02) despite the relatively small number of events, which suggests that the optimal time for IMC is in the first month of life. That IMC is safer at younger ages is consistent with the few published reports on this topic [30], [31] and reinforces the WHO recommendation that IMC be performed within the first two months of life [32].

Early adverse events following neonatal or infant circumcision are generally minor and treatable. These include: bleeding resolved with conservative management or suturing, minor infection resolved with antibiotic therapy, pain that can be managed with analgesic therapy, problems with incomplete separation or retention of Plastibell or other disposable device used in circumcision, and parental dissatisfaction with appearance [33], [34]. The AEs we observed in this study are consistent with these early, minor events. More rarely, severe or life threatening adverse events following IMC have been reported. These complications can be early or late and include: complete denudation of penile shaft skin, formation of skin bridges between the shaft and glans, damage to the penis including partial or complete amputation or necrosis, damage to the urethra, buried or trapped penis and meatal stenosis [34]–[37]. We did not actively follow infants after the initial review, usually occurring within one week of the procedure. Therefore, we cannot exclude the possibility of serious late AEs. However, we did not receive telephone calls or follow-up visits from parents with concerns about late complications and we offered treatment of adverse events free of charge.

This analysis has several limitations. We did not record information about the number or type of phone calls between study staff and parents who had questions or concerns about IMC. Our follow-up period was relatively short and the routine monitoring data did not collect several variables of interest potentially associated with AE risk (e.g. hygiene practices). Nevertheless, this study provides valuable data on AE rate and type in an area where IMC is little-practiced and relatively few published reports on this topic exist.

The rate at which non-research parents brought their infant back for the three to four day post-surgical review was low at 41%. However, the AE rate among non-research infants was comparable to that among research infants (2.9% vs. 2.4%), and the latter group had a 95% follow-up rate. Review rates for IMC may be low in the absence of financial reimbursement for transport costs and active follow-up.

Our study shows IMC services can be provided safely by nurses and clinical officers and with high parental satisfaction in a developing country setting where infant circumcision is little-known and rarely practiced. These results are consistent with the two-month cut-off period recommended by the WHO, and suggest that the optimal time for IMC is the first month of life. Targeted efforts should be made to encourage parents to bring their children for IMC within the first month of life. Our findings are informative for policy makers who seek to scale-up IMC services for long-term, sustainable HIV prevention and for circumcision's other health benefits.
